# Low MAD2 expression levels associate with reduced progression-free survival in patients with high-grade serous epithelial ovarian cancer

**DOI:** 10.1002/path.3035

**Published:** 2012-01-17

**Authors:** Fiona Furlong, Patricia Fitzpatrick, Sharon O'Toole, Sine Phelan, Barbara McGrogan, Aoife Maguire, Anthony O'Grady, Michael Gallagher, Maria Prencipe, Aloysius McGoldrick, Paul McGettigan, Donal Brennan, Orla Sheils, Cara Martin, Elaine W Kay, John O'Leary, Amanda McCann

**Affiliations:** 1 UCD School of Medicine and Medical Science (SMMS), UCD Conway Institute of Biomolecular and Biomedical Research, University College DublinBelfield, Dublin 4, Ireland; 2 UCD School of Public Health, Physiotherapy and Population Science, University College DublinBelfield, Dublin 4, Ireland; 3 Departments of Histopathology and Obstetrics and Gynaecology, Trinity College DublinDublin 2, Ireland; 4 Department of Pathology, Royal College of Surgeons in Ireland (RCSI), Beaumont HospitalDublin, Ireland; 5 Department of Pathology, The Coombe Women and Infants University Hospital & Trinity College DublinDublin 8, Ireland

**Keywords:** miR-433, MAD2, chemoresistance, epithelial ovarian cancer, paclitaxel

## Abstract

Epithelial ovarian cancer (EOC) has an innate susceptibility to become chemoresistant. Up to 30% of patients do not respond to conventional chemotherapy [paclitaxel (Taxol®) in combination with carboplatin] and, of those who have an initial response, many patients relapse. Therefore, an understanding of the molecular mechanisms that regulate cellular chemotherapeutic responses in EOC cells has the potential to impact significantly on patient outcome. The mitotic arrest deficiency protein 2 (MAD2), is a centrally important mediator of the cellular response to paclitaxel. MAD2 immunohistochemical analysis was performed on 82 high-grade serous EOC samples. A multivariate Cox regression analysis of nuclear MAD2 IHC intensity adjusting for stage, tumour grade and optimum surgical debulking revealed that low MAD2 IHC staining intensity was significantly associated with reduced progression-free survival (PFS) (*p* = 0.0003), with a hazard ratio of 4.689. The *in vitro* analyses of five ovarian cancer cell lines demonstrated that cells with low MAD2 expression were less sensitive to paclitaxel. Furthermore, paclitaxel-induced activation of the spindle assembly checkpoint (SAC) and apoptotic cell death was abrogated in cells transfected with MAD2 siRNA. *In silico* analysis identified a miR-433 binding domain in the MAD2 3′ UTR, which was verified in a series of experiments. Firstly, MAD2 protein expression levels were down-regulated in pre-miR-433 transfected A2780 cells. Secondly, pre-miR-433 suppressed the activity of a reporter construct containing the 3′-UTR of MAD2. Thirdly, blocking miR-433 binding to the MAD2 3′ UTR protected MAD2 from miR-433 induced protein down-regulation. Importantly, reduced MAD2 protein expression in pre-miR-433-transfected A2780 cells rendered these cells less sensitive to paclitaxel. In conclusion, loss of MAD2 protein expression results in increased resistance to paclitaxel in EOC cells. Measuring MAD2 IHC staining intensity may predict paclitaxel responses in women presenting with high-grade serous EOC. Copyright © 2012 Pathological Society of Great Britain and Ireland. Published by John Wiley & Sons, Ltd.

## Introduction

Epithelial ovarian cancer (EOC) is the most lethal gynaecological malignancy worldwide, with most patients diagnosed with advanced disease at primary presentation 1. Cytoreductive surgery followed by a combination of platinum based and paclitaxel (Taxol®) chemotherapy is the first line treatment strategy currently in use. Most advanced stage patients respond to this treatment modality 2. However, 70% of women will relapse and the 5 year survival rate is < 25% for patients diagnosed with stage III–IV EOC 2. Cellular chemotherapeutic resistance is a major contributor to poor patient response and reduced overall survival in patients with ovarian tumours 3. Therefore, an understanding of the molecular mechanisms that regulate cellular chemotherapeutic responses has the potential to impact significantly on patient outcome.

The taxane, paclitaxel, is a microtubule-stabilizing agent that functions primarily by interfering with the spindle microtubule dynamics causing cell cycle arrest and apoptosis 4. Cell cycle arrest is achieved by the activation of the spindle assembly checkpoint (SAC), resulting in cellular arrest in the G_2_–M phase of the cell cycle. Specifically the SAC ensures the correct alignment and segregation of chromosomes into daughter cells exiting mitosis 5. Microtubule dynamics are essential to this process and appropriate tension across the mitotic spindle is required to silence the SAC, which causes sister chromatids to separate, thereby facilitating mitosis 6. In the presence of mitotic defects, cell death can be triggered during mitosis or after the cell has exited mitosis 7. Chemoresistance to paclitaxel can be attributed to functional aberrations in a variety of molecular pathways governing cell cycle progression, growth promotion and the activation of apoptosis, resulting in poor patient response to paclitaxel-based regimens 4.

The mitotic arrest deficiency protein 2 (MAD2) is a key regulator of the SAC pathway 8. MAD2 protein expression is relatively unchanged throughout the cell cycle with SAC activation occurring through the association of MAD2 with MAD1 and the APC complex (APC/C) 9, 10. The cell cycle-regulated phosphorylation of MAD2 modulates a conformational change in MAD2 that causes MAD1 and APC/C binding 10. Importantly, reduced MAD2 protein expression results in attenuated SAC responses in a number of cell models 11, 12 and subsequent drug resistance to paclitaxel 13–15. Although reduced MAD2 expression has been reported in human cancer 16, 17, the mechanisms underlying MAD2 down-regulation remain elusive. Importantly, mutations in the *MAD2* gene are reportedly rare in human cancer 18, 19 and we have previously shown that down-regulation of MAD2 in cancer is not associated with promoter hypermethylation 20.

In this study we have examined the immunohistochemical levels of MAD2 protein expression in 82 patient samples from a series of 45 full-face formalin-fixed, paraffin-embedded (FFPE) high-grade serous EOC sections of similar stage and grade and in a separate cohort of 37 high-grade serous EOC samples represented on a previously published tissue microarray (TMA) platform 21. Our *in vitro* analyses have demonstrated a central role of MAD2 in the activation of mitotic cell death by paclitaxel. Moreover, we report for the first time the down-regulation of MAD2 by the microRNA, miR-433, which results in an attenuated cellular response to paclitaxel.

## Materials and methods

### Patients, tissue specimens and histopathological data

A total of 82 patients from two independent patient cohorts were analysed in this study. Full-face paraffin-embedded sections from 45 high-grade serous carcinoma specimens were obtained from patients undergoing laparotomy for ovarian cancer in St. James's Hospital, Dublin, Ireland (patient cohort 1). Informed consent was obtained from each patient by the research team prior to surgery, with ethical approval received from the St. James's Hospital/the Adelaide and Meath Hospital, Dublin, incorporating the National Children's Hospital Research Ethics Committee, Dublin, Ireland. Tumours were staged according to the International Federation of Gynecology and Obstetrics (FIGO) staging system. Clinicopathological details of the specimens are shown in Table S1 (see Supporting information). All patients received adjuvant carboplatin/paclitaxel chemotherapy.

Thirty-seven additional cases of high-grade serous EOC (patient cohort 2), which had been part of a previous study, were also analysed 21. Clinicopathological details of the specimens are shown in Table S1 (see Supporting information). All patients were treated with platinum-based therapy alone or paclitaxel and platinum.

Patient charts were monitored retrospectively and follow-up information was available including age, optimum surgical debulking (< 1 cm) and time to recurrence. Progression-free survival (PFS) for each patient was calculated from the date the sample was taken to the date of recurrence.

### MAD2 Immunohistochemistry (IHC)

Five µm sections were immunostained with the MAD2 monoclonal antibody (BD Transduction Laboratories) at a dilution of 1:100 on an automated platform (Bond™ system, Leica MicroSystems™, Newcastle, UK), as previously described 20.

### Manual MAD2 IHC scoring

IHC expression was quantified in patient cohort 1 (*n* = 45 full-face sections) and in patient cohort 2 (*n* = 37 on a TMA) by two independent observers (SP and AM, SP and BM, respectively). The full-face sections and the TMA cores were scored manually, based on intensity of nuclear staining (1+, negative; 2+, weak; 3+, moderate; and 4+, strong). In cases where the two observers differed, slides were re-evaluated jointly by both observers and a consensus was reached. The poor quality of one set of ovarian tumour cores resulted in its exclusion from subsequent analyses.

### Tissue culture

The ovarian cancer cell lines, ovca432 and ovca433 were a kind gift from R. Bast (UT-MD Anderson Cancer Center and the Dana Faber Cancer Institute, USA). The ovarian cancer cell lines were routinely cultured in minimal essential medium (MEM; Sigma) supplemented with 10% v/v FCS, 100 µg streptomycin, 100 U/ml penicillin, 0.3 mg/ml glutamine and 1% sodium pyruvate. UPN251, ovcar7 and A2780 ovarian cancer cell lines were a kind gift from Robert F Ozols (Fox Chase Cancer Center, USA). These cell lines were cultured in RPMI 1640 supplemented with 10% v/v fetal calf serum (FCS), 100 µg streptomycin, 100 U/ml penicillin and 0.3 mg/mL glutamine. The cell lines were maintained at 37 °C in a humidified incubator containing 5% CO_2_ and were routinely tested and shown negative for mycoplasma contamination.

### RNA extraction

The 45 full-face FFPE high-grade serous ovarian carcinoma sections (patient cohort 1) were haematoxylin and eosin (H&E)-stained and pathologically reviewed (SP). Where tumour cell density was > 90%, whole sections were taken for miRNA analysis. In cases where a tumoural stroma component was significantly present, areas were macro-dissected to enrich for the epithelial population. Total RNA was isolated using the RNeasy FFPE Kit (Qiagen). For the cell lines, RNA was extracted using the guanidine-based TRIzol™ reagent according to the manufacturer's instructions.

### qRT–PCR (quantitative real-time PCR; TaqMan®)

MAD2 and hsa-miR-433 were sourced from ABI as Assay-On-Demand gene expression assays and total RNA analysed by qRT–PCR on the ABI PRISM 7900 Sequence Detection System; mRNA levels were determined using the standard curve method (ABI Prism 7700 Sequence Detection System, User Bulletin No. 2) and quantified using the TaqMan® comparative CT method of analysis, as previously described 20.

### Transient transfections

Pre-miR-433 and scrambled control microRNAs were purchased from Applied Biosystems. MAD2 siRNA and scrambled controls were purchased from Dharmacon and MAD2 morpholino and scrambled morpholino oligos were sourced from Gene-tools^©^. 400 nm pre-MiRs, 100 nm siRNA or 10 µM morpholino oligos were incubated with Hiperfect (Qiagen) for 20 min at room temperature and mixed into a cell suspension (3.6 × 10^5^ cells). Cells were then plated onto six- and 96-well plates and maintained in the presence of transfection mix for 2 h before changing the medium to full growth medium. The following day, the cells were treated with 10 and 50 nm paclitaxel for 24 h.

### SDS–PAGE and western blotting

Whole cell lysates were prepared using RIPA buffer. Samples were normalized for total protein, resuspended in reducing sample buffer and separated by SDS–PAGE 22. Proteins were then electrophoretically transferred to a nitrocellulose membrane (100 V for 60 min) and analysed with antibodies raised against MAD2 (BD Transduction Laboratories), cyclin B1, poly(ADP–ribose) polymerase (PARP), phospho (ser10)-Histone H3, cleaved caspase 3 (Cell Signaling) and β-actin (Sigma). Horseradish peroxidase-conjugated secondary antibodies (DakoCytomation) were used in conjunction with a ECL chemiluminescence detection system (Pierce).

### Cell viability assay

Ovarian cancer cells treated with paclitaxel (10 and 50 nm) for 24 and 48 h were analysed by the MTT assay, as previously described 20.

### Luciferase reporter assay

The pMIR–REPORT miRNA expression reporter vector system (Applied Biosystems) was used to construct a reporter plasmid containing 500 bp of the *MAD2* mRNA transcript containing the putative miR-433-binding domain in the 3′ UTR, according to the manufacturer's instructions, with primers shown in Table 2 (see Supporting information). Cells were transfected with pre-miR-433 or scrambled control pre-miRs, as outlined above. After 24 h, the cells were co-transfected with the MAD2–pMIR–REPORT and TK *Renilla* reporter plasmids. MAD2–pMIR–REPORT luciferase activity was measured as previously des- cribed 23.

### Statistical analyses

All data are presented as mean ± SE for at least three independent experiments. Data analysis was performed using Microsoft Excel and SAS, version 9 (SAS Institute, Cary, NC, USA). Student's *t*-test was used for comparison of means and Kaplan–Meier survival curves with log rank tests are shown for PFS, based on IHC staining intensity 1+ and 2+ compared to 3+ and 4+. The effect of stage, tumour grade and optimum debulking on MAD2 IHC staining intensity was assessed in a multivariate Cox regression analysis.

## Results

### Low MAD2 nuclear staining intensity is associated with reduced PFS in high-grade serous EOC post-chemotherapy

**Figure 1:**
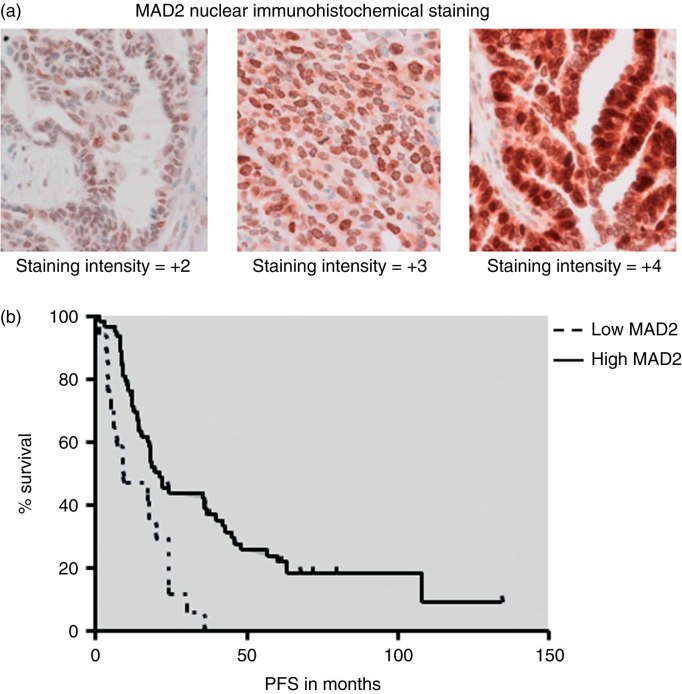
Nuclear MAD2 IHC staining intensity is associated with progression-free survival (PFS) in high-grade serous EOC. (a) Full-face FFPE sections demonstrating IHC staining intensity for MAD2, low (2+), intermediate (3+), high (4+). (b) Multivariate Cox's regression hazard analysis: correlation of low MAD2 IHC staining intensity expression with progression-free survival (PFS) in patients with high-grade serous EOC. Multivariate Cox's regression hazard analysis (adjusted for stage, tumour grade and optimum surgical debulking < 1 cm) showed a significant correlation between low MAD2 IHC staining intensity and PFS (*p* = 0.0003; HR 4.689)

A total of 82 patient samples comprising of 45 full-face FFPE high-grade serous ovarian carcinoma sections and a TMA representing an additional 37 FFPE tumour cores were immunohistochemically stained for MAD2. MAD2 stained the nucleus of ovarian epithelial cancer cells with varying degrees of intensity (Figure 1a). In addition, MAD2 negative and positive cancer cells were evident in the same tumour (Figure 1a). The Kaplan–Meier survival curve for MAD2 IHC staining intensity and PFS demonstrated that low MAD2 IHC intensity is significantly associated with a reduced PFS [log rank *p* = 0.0005; hazard ratio (HR) 2.75] (Figure 1b). In a multivariate Cox regression analysis adjusting for stage, grade and optimum surgical debulking, the association between low MAD2 IHC and PFS remained independently statistically significant (log rank *p* = 0.0003), with an increased HR of 4.689.

### Ovarian cancer cell lines expressing high levels of MAD2 are more sensitive to paclitaxel

**Figure 2:**
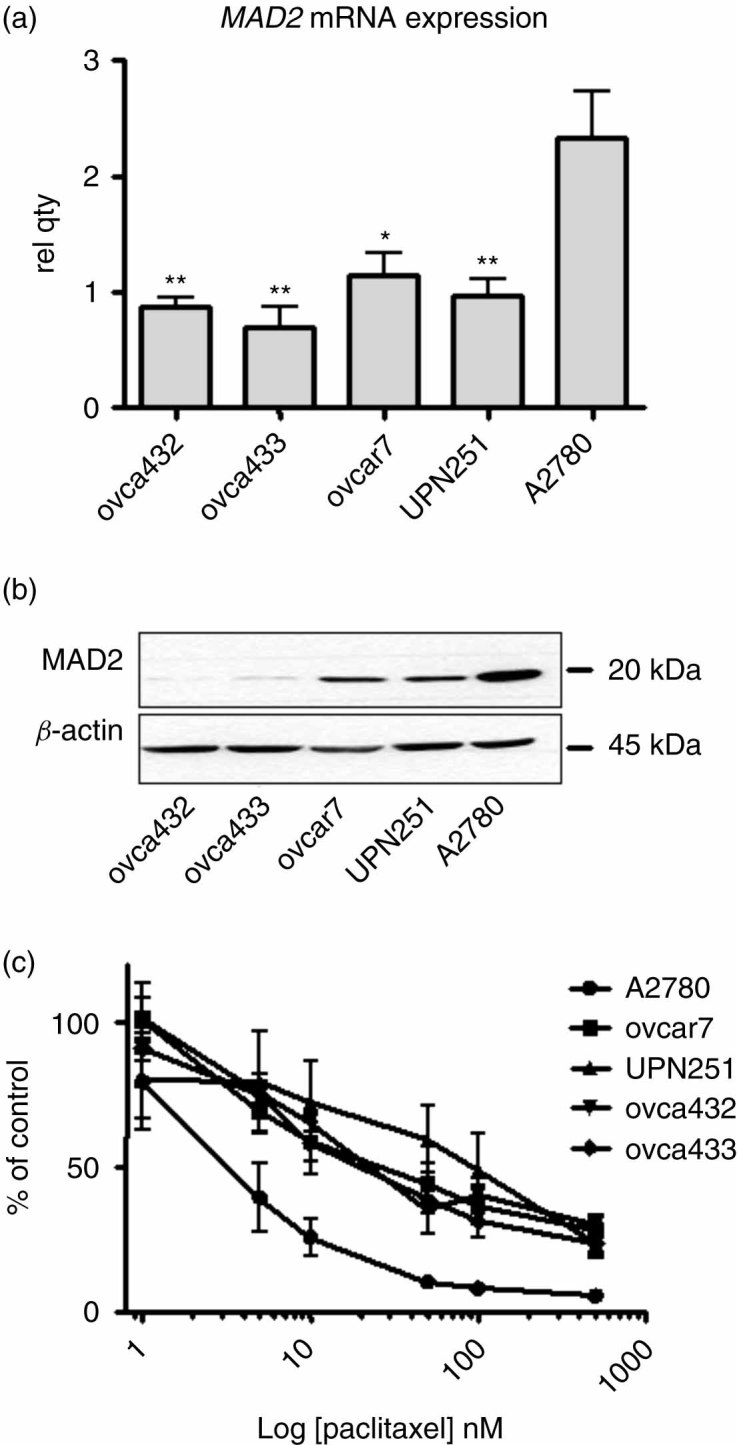
Ovarian cancer cells with low MAD2 protein expression are less sensitive to paclitaxel. (a) Bar graph of qRT–PCR for *MAD2* mRNA expression. Relative expression levels compared to A2780 cells were determined using the standard curve method (ABI Prism 7700 Sequence Detection System, User Bulletin no. 2) and quantified using the TaqMan® comparative CT method of analysis. (b) Western blot analyses for MAD2 protein expression and western blot for β-actin loading control. (c) Line graph representing a dose–response curve for cell viability assessed in a MTT assay in ovarian cancer cells treated with 1–500 nm paclitaxel for 48 h. The data presented are representative of at least three independent experiments. Student's *t*-test was used for comparison of means (*p < 0.05, **p < 0.01 and ***p < 0.001)

MAD2 expression was assessed in five EOC cell lines by qRT–PCR (Figure 2a) and western blot analyses (Figure 2b). *MAD2* mRNA (Figure 2a) and protein levels (Figure 2b) were greatest in the A2780 EOC cell line. The A2780 cell line was previously shown to be sensitive to paclitaxel at a concentration of 1.4 nm 24. The dose–response curve for loss of cell viability at 48 h demonstrated that A2780 cells were more sensitive to paclitaxel compared to the other cell lines (Figure 2c). However, MAD2 expression levels and the effects of paclitaxel on cellular viability in the EOC cells are not linear. Specifically, MAD2 expression in UPN251 and ovcar7 is higher than MAD2 expression in ovca432 and ovca433 (Figure 2a, b). This, however, does not result in a greater loss of cell viability in response to paclitaxel (Figure 2c).

**Figure 3:**
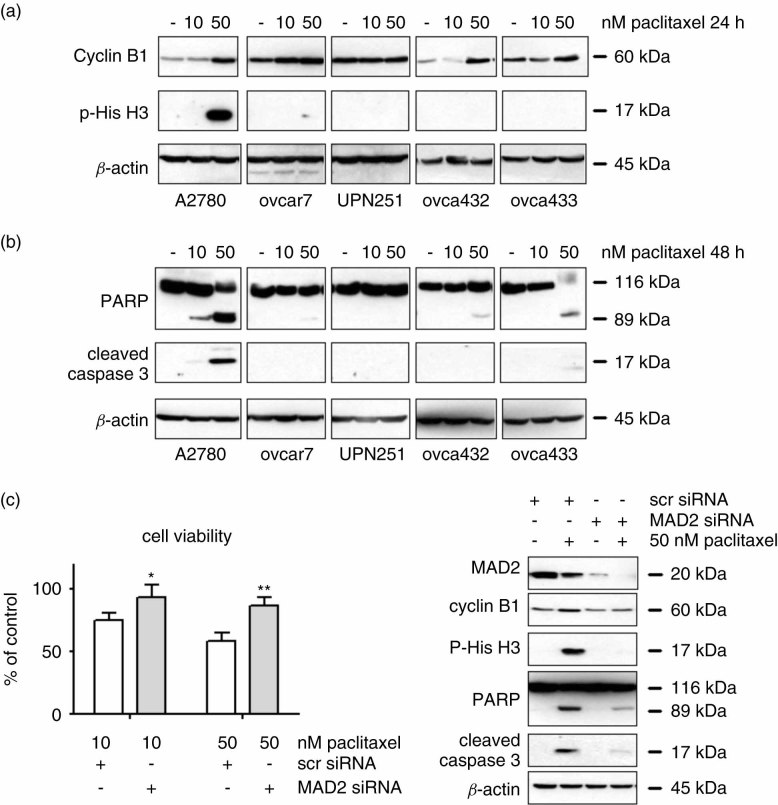
Cellular responses to paclitaxel are compromised in ovarian cells that express low levels of MAD2. (a) Western blot analyses for cyclin B1, phospho(Ser10)-Histone H3 and PARP cleavage in the ovarian cancer cell lines treated with 10 and 50 nm paclitaxel for 24 h. (b) Western blot analyses for PARP and cleaved caspase 3, in the ovarian cancer cell lines treated with 10 and 50 nm paclitaxel for 48 h. A western blot for β-actin was used as a loading control. The data presented are representative of at least three independent experiments. (c) MTT viability assay of A2780 cells transiently transfected with MAD2 siRNA and scrambled controls and treated with paclitaxel for 24 h. Western blot analyses of A2780 cells transiently transfected with MAD2 siRNA and scrambled controls and treated with paclitaxel for 24 h. Data presented are representative of at least three independent experiments (*p < 0.05, **p < 0.01)

To determine whether the reduction of cell viability measured in Figure 2c may reflect the anti-proliferative action or the apoptotic action of paclitaxel, we investigated paclitaxel activation of the SAC and induced mitotic arrest by western blot analyses for cyclin B1 stabilization and phospho(Ser10)-Histone H3 (Figure 3a) 25, 26. Paclitaxel-induced apoptosis was assessed by western blot analyses for PARP and cleaved caspase 3 (Figure 3b) 27. The treatment of A2780 cells with 50 nm paclitaxel for 24 h induced cyclin B1 stabilization and phosphorylation of serine 10 on Histone H3, indicating the activation of the SAC and a sustained mitotic arrest (Figure 3a). Conversely, the action of 50 nm paclitaxel for 24 h resulted in a diminished cyclin B1 stabilization in ovcar7, ovca432 and ovca433 cells when compared with A2780 cells and failed to cause mitotic arrest in these cells, evidenced by the absence of Histone H3 phosphorylation at 24 h (Figure 3a) and 48 h (data not shown). Paclitaxel did not cause cyclin B1 stabilization or Histone H3 phosphorylation in UPN251 cells, indicating additional defects in activation of the SAC response in this cell line (Figure 3a). Cyclin B1 stabilization and Histone H3 phosphorylation was not detected in any of the cell lines treated with 10 and 50 nm paclitaxel for 48 h (data not shown).

Activation of the SAC which resulted in a sustained mitotic arrest by paclitaxel in A2780 cells (Figure 3a) also resulted in PARP and caspase 3 cleavage at 24 h (data not shown) and 48 h (Figure 3b). These data demonstrate the activation of paclitaxel-induced cell death during mitosis, as previously described 15. Paclitaxel failed to cause significant PARP and caspase 3 cleavage in the other EOC cell lines. This suggests that activation of apoptosis during mitosis may be defective in these cell lines as a result of reduced MAD2 expression.

To further investigate the role of MAD2 in the activation of mitotic cell death in response to paclitaxel, MAD2 expression was knocked down in the A2780 cells by siRNA (Figure 3c). MAD2 knockdown cells were more viable in the presence of 10 and 50 nm paclitaxel compared to scrambled siRNA control cells (Figure 3c, left). Moreover, MAD2 knockdown by siRNA abolished paclitaxel induced SAC activation and mitotic arrest, as demonstrated by the absence of cyclin B1 stabilization and Histone H3 phosphorylation (Figure 3c, right) in MAD2 siRNA-transfected cells. The activation of the apoptotic response resulting in PARP and caspase 3 cleavage was also inhibited in the MAD2 siRNA-transfected cells compared to scrambled control transfected cells (Figure 3c, right). Overall, these results have demonstrated that paclitaxel-induced SAC activation and the subsequent activation of apoptosis during mitosis in ovarian cancer cells is associated with high MAD2 protein expression.

### Pre-miR-433 causes down-regulation of MAD2 protein expression and attenuates the cellular responses to paclitaxel in ovarian cancer cells

**Figure 4:**
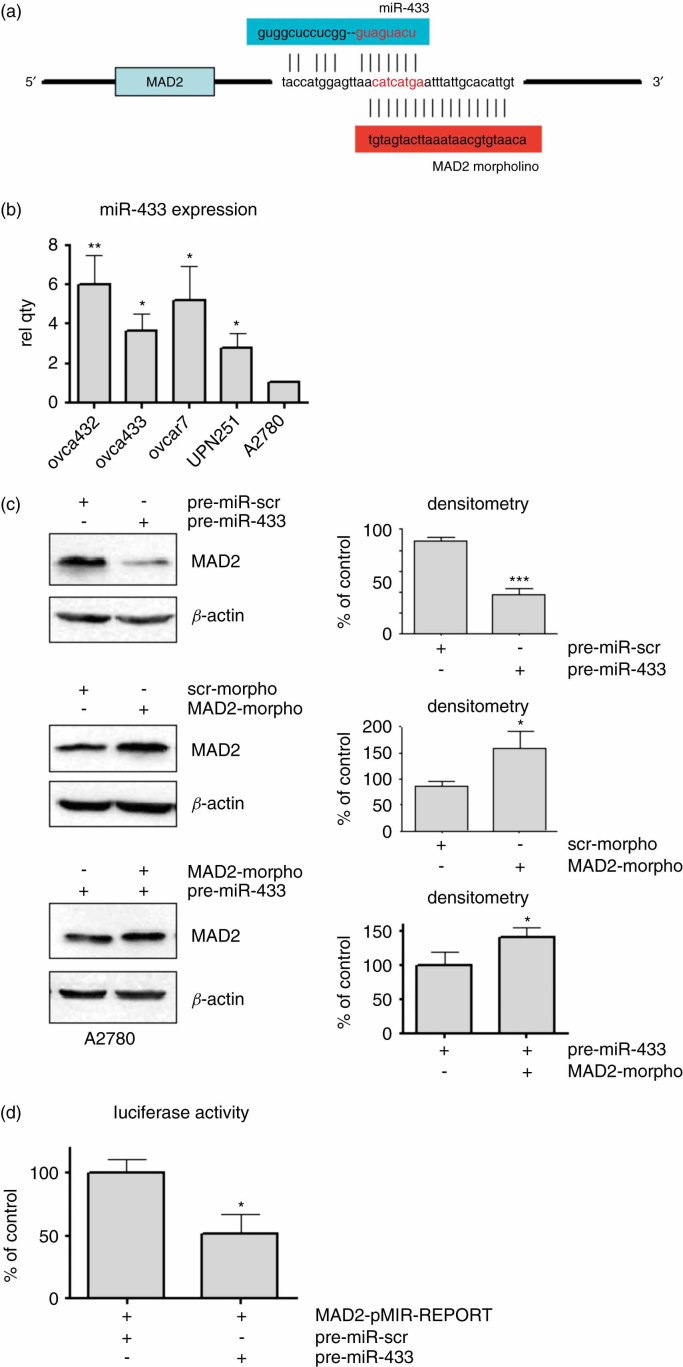
Binding of miR-433 to the 3′ UTR of *MAD2* mRNA down-regulates MAD2 protein expression. (a) Schematic representation of the miR-433 binding region in the MAD2 3′ UTR and the sequence of the MAD2 morpholino oligonucleotide complimentary to the miR-433-MAD2 binding domain. (b) Bar graph of relative miR-433 gene expression in the ovarian cancer cell lines. Relative expression levels compared to A2780 cells were determined using the standard curve method (ABI Prism 7700 Sequence Detection System, User Bulletin No. 2) and quantified using the TaqMan® comparative CT method of analysis. (c) MAD2 and β-actin-loading control western blot analyses of A2780 cell lines transfected with pre-miR-433 and scrambled controls or MAD2 morpholino and morpholino scrambled controls. Bar graphs represent MAD2 expression as a percentage of control measured by densitometry using ImageJ. The data presented are representative of at least three independent experiments. (d) Comparison of normalized luciferase activity in A2780 cells transfected with MAD2–pMIR–REPORT and pre-miR-scr or pre-miR-433. Student's *t*-test was used for comparison of means (*p < 0.05, **p < 0.01 and ***p < 0.001)

MicroRNAs (miRs) are a large family of gene regulators, which constitute a novel class of non-coding RNA that bind to mRNA and result in either the degradation or inhibition of translation of their target genes into protein 28. *In silico* analysis using MicroCosm (previously mirBase) 29 demonstrated a putative miR-433 microRNA binding domain in the 3′ UTR of the MAD2 transcript (Figure 4a). miR-433 expression was assessed in the five ovarian cancer cell lines by qRT–PCR (Figure 4b). The ovarian cancer cell line A2780, which expressed the highest level of *MAD2* mRNA (Figure 2a) and protein expression levels (Figure 2b), displayed the lowest miR-433 expression levels (Figure 4b). When comparing the A2780 cell line with the other ovarian cancer cell lines, lower *MAD2* mRNA and protein levels (Figure 2a, b) were associated with concomitant higher miR-433 expression levels (Figure 4b). Subsequently, we transiently transfected A2780 cells with the 70 bp precursor microRNA, pre-miR-433 (Figure 4c, top). Pre-miR-433 resulted in the down-regulation of MAD2 protein expression compared with the scrambled control pre-miRs (Figure 4c, top), which lasted for 72 h in HeLa cells (see Supporting information, Figure S1a).

To investigate whether miR-433 binds to the MAD2 3′ UTR, we designed a morpholino oligonucleotide targeting the putative MAD2-miR-433-binding domain (Figure 4a). The transient transfection of the MAD2 morpholino oligonucleotide caused an increase in MAD2 protein expression (Figure 4c, middle) and we conclude that the MAD2 morpholino oligonucleotide protects from endogenous miR-433 by sterically hindering miR-433 from binding to the MAD2 3′ UTR. Finally, MAD2 protein expression was analysed in A2780 cells that were co-transfected with pre-miR-433 and the MAD2 morpholino oligonucleotide and compared to cells transfected with pre-miR-433 alone. The western blot analyses revealed that MAD2 protein expression was higher in the presence of the MAD2 morpholino oligonucleotide (Figure 4c, bottom).

Finally, the previously described pMIR–REPORT miRNA expression reporter vector system 30 for measuring mRNA–microRNA interactions was also used to investigate whether miR-433 interacts with the MAD2 3′ UTR. Co-transfection of pre-miR-433 with pMIR–REPORT vector containing the MAD2 3′ UTR caused marked reduction in luciferase activity compared to scrambled control pre-miRs (Figure 4d). Overall, these experiments demonstrate a functional interaction between miR-433 and MAD2 3′ UTR. Furthermore, blocking this binding site protects MAD2 from miR-433 induced protein down-regulation.

### The transient transfection of pre-miR-433 attenuates paclitaxel responses in ovarian cancer cells

**Figure 5:**
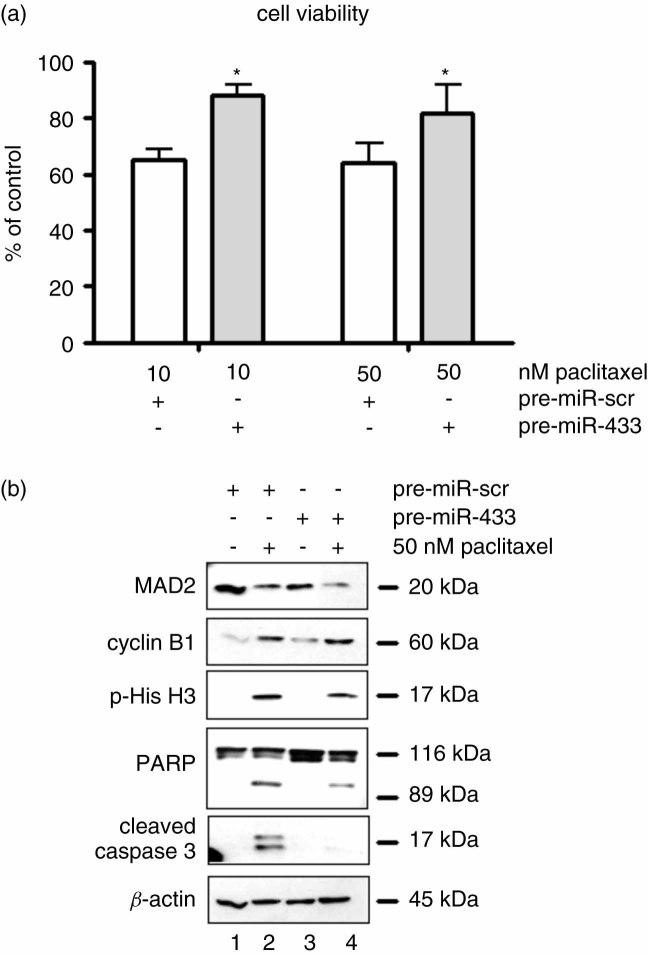
Transient transfection of pre-miR-433 compromises paclitaxel responses in A2780 cells. (a) Bar graph representing the relative viability assessed by MTT assay in A2780 cells transiently transfected with pre-miR-433 and treated with 10 and 50 nm paclitaxel compared to scrambled control. (b) Western blot analyses for MAD2, cyclin B1, phospho(Ser10)–Histone H3, PARP cleavage and cleaved caspase 3 in A2780 cells transiently transfected with scrambled control microRNAs and pre-miR-433. The cell lines were treated with 10 and 50 nm paclitaxel for 24 h. A western blot for β-actin was used to demonstrate loading. The data presented are representative of at least three independent experiments. Mean differences were calculated by Student's *t*-test, where statistical significance was assessed as *p < 0.05

Cellular responses to paclitaxel were investigated in A2780 cells transfected with pre-miR-433 by the MTT cell viability assay and western blot analyses. The down-regulation of MAD2 by pre-miR-433 resulted in a decreased sensitivity to 10 and 50 nm paclitaxel, as judged by the MTT assay (Figure 5a). The activation of the SAC and apoptosis in response to paclitaxel was also compromised in A2780 cells transfected with pre-miR-433 (Figure 5b). Specifically, miR-433 induced down-regulation of MAD2 protein expression, resulted in paclitaxel induced cyclin B1 stabilization (Figure 5b). However, phosphorylation of Histone H_3_, PARP cleavage and cleaved caspase 3 levels were attenuated following treatment with paclitaxel compared to scrambled controls (Figure 5b; cf lane 2 with lane 4). Finally, the expression of miR-433 was analysed by RT–PCR and normalized to U6 in patient cohort 1 (*n* = 45). The median expression level was 1.07. For statistical analysis, miR433 was grouped by tertiles, as follows: low (< 0.7); intermediate (range 0.7–1.8); and high (> 1.8) miR-433 expression. The Kaplan–Meier survival curve and log rank analysis revealed that high miR-433 was non-significantly associated with reduced PFS (*p* = 0.078) (see Supporting information, Figure S2).

## Discussion

The role of aberrant MAD2 expression and cellular drug resistance to chemotherapy agents has been studied extensively in cell culture models 31–34. However, there are few published studies on human clinical cancer specimens. In this manuscript we have demonstrated the novel association between low MAD2 IHC staining intensity and reduced PFS in high-grade serous EOC. Moreover, from the observation that MAD2-positive ovarian epithelial cells co-exist with MAD2-negative ovarian cancer cells within the same tissue specimen, we hypothesize that the MAD2-negative ovarian epithelial cells are chemoresistant, which may explain the high propensity for EOC to recur. Indeed, data presented in this study demonstrated that paclitaxel can no longer induce cell cycle arrest or apoptosis in ovarian cells devoid of MAD2. While paclitaxel has provided the biggest single advance in the management of EOC over the last decade 35, paclitaxel is administered in combination with platinum-based chemotherapy. Therefore, the apparent prognostic value of MAD2 IHC staining documented in this study cannot be attributable to paclitaxel sensitivity alone. Specifically, EOC is routinely stratified by chemoresponse to cisplatin 36. As MAD2 was previously reported to mediate cellular responses to DNA-damaging agents in cancer cells 33, measuring MAD2 IHC staining intensity may also be predictive of chemoresponse to cisplatin in EOC.

Loss of BRCA1 significantly contributes to the pathogenesis of sporadic EOC and tumours with inactive BRCA1 are reported to have a better outcome, due to improved responses to cisplatin 37. Therefore, tumours with high MAD2 IHC intensity and very long PFS are likely to be highly enriched for BRCA1 inactivation. Conversely, loss of BRCA1 function is reported to be predictive of resistance to the taxanes 35. BRCA1 has been found to transcriptionally regulate the expression of MAD2 38. However, the degree of BRCA1 inactivation reportedly associated with EOC does not correlate with the high levels of MAD2 IHC measured in our study, and other factors must also regulate MAD2 expression in EOC. A recent study by Schvartzman *et al* 39 describes a tumour model where MAD2 becomes up-regulated as a result of P53 inactivation resulting in chromosomal instability (CIN). P53 is frequently inactivated in EOC but its use as a biomarker of chemoresponse in EOC is contentious 1, 35. While it is not yet clear why MAD2 may be independently predicting PFS, we have demonstrated that MAD2 expression is required to mediate chemoresponses in EOC cells and MAD2-negative cells are likely to be chemoresistant.

How MAD2 expression becomes down-regulated by a microRNA was also demonstrated in our study and we report for the first time the down-regulation of MAD2 protein expression by miR-433. MiR-433 exists in a small cluster of five microRNAs that are located in the human imprinted 14q32 domain 40. Furthermore, expression of microRNAs from this cluster is reported to be altered in cancer by epigenetic silencing 41, 42. We propose that miR-433 expression becomes deregulated in ovarian cancer resulting in the down-regulation of MAD2 expression. We hypothesize that this contributes to a poorer chemoresponse in EOC as paclitaxel responses are attenuated in pre-miR-433 transfected cells as a result of MAD2 protein down-regulation.

In summary, this study describes a micro-RNA-mediated mechanism by which ovarian cancer cells can become less sensitive to paclitaxel. We suggest that profiling MAD2 immunohistochemical staining intensity could predict chemoresponse in women presenting with high-grade serous EOC. Moreover, understanding chemoresponses in ovarian tumours will lead to improved patient management and treatment options for women diagnosed with this disease.

## SUPPORTING INFORMATION ON THE INTERNET

The following supporting information may be found in the online version of this article:

**Figure S1.** MAD2 western blot analysis of HeLa cells transiently transfected with pre-miR-433 and scrambled controls.

**Figure S2.** Univariate Kaplan–Meier survival curve: correlation of miR-433 expression with progression-free survival (PFS).

**Table S1.** Clinicopathological details of ovarian specimens.

**Table S2.** Sequence of primers for cloning the MAD2 3′ UTR.
