# Supplementary figures and images for: Low MAD2 expression levels associate with reduced progression-free survival in patients with high-grade serous epithelial ovarian cancer
Source: J Pathol. 2012 Jan 17;226(5):746–55. doi: 10.1002/path.3035 (PMC3593171; doi:10.1002/path.3035)

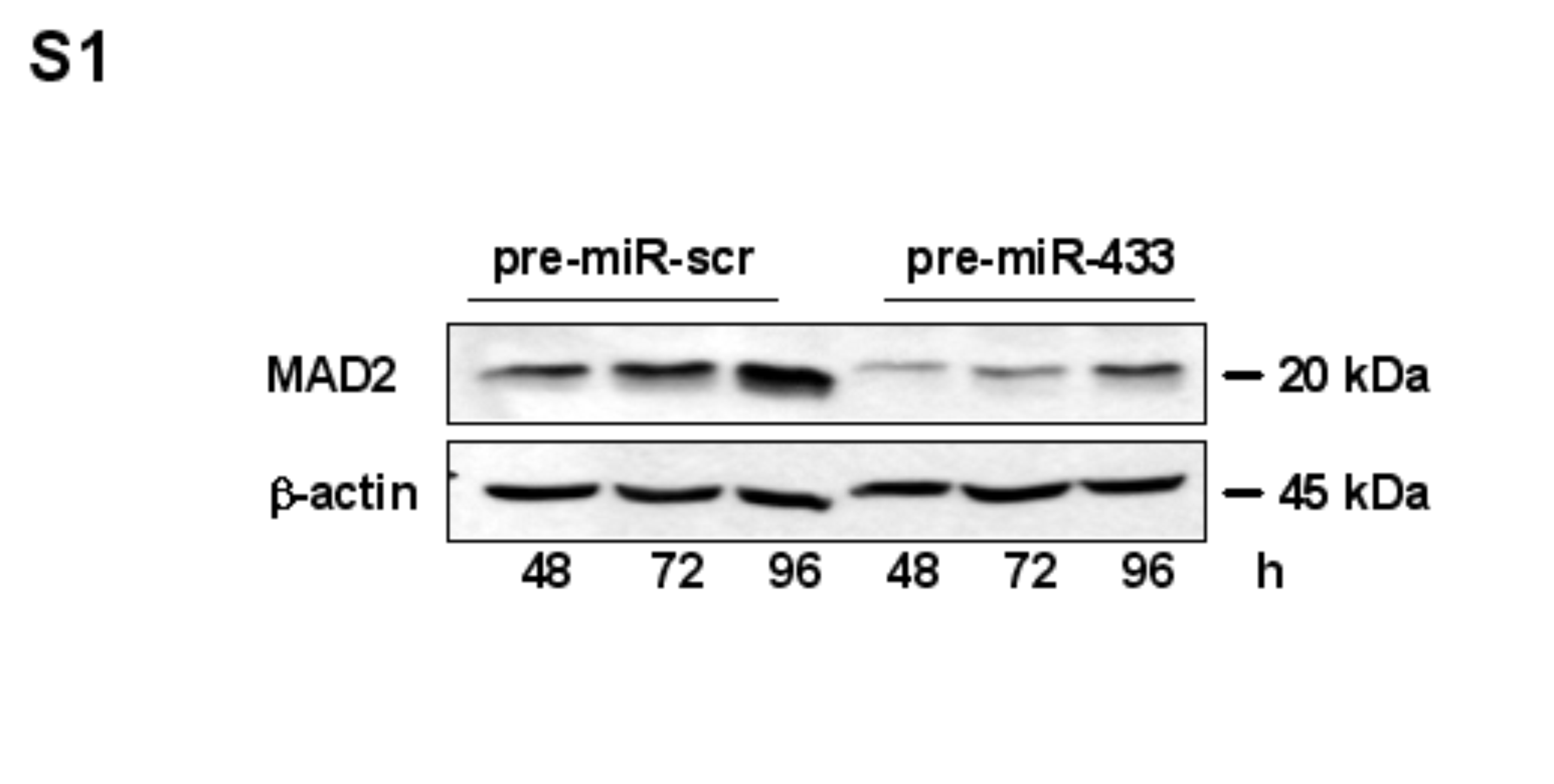

Supplement: Supplementary file 1 [file path0226-0746-SD1.tif]

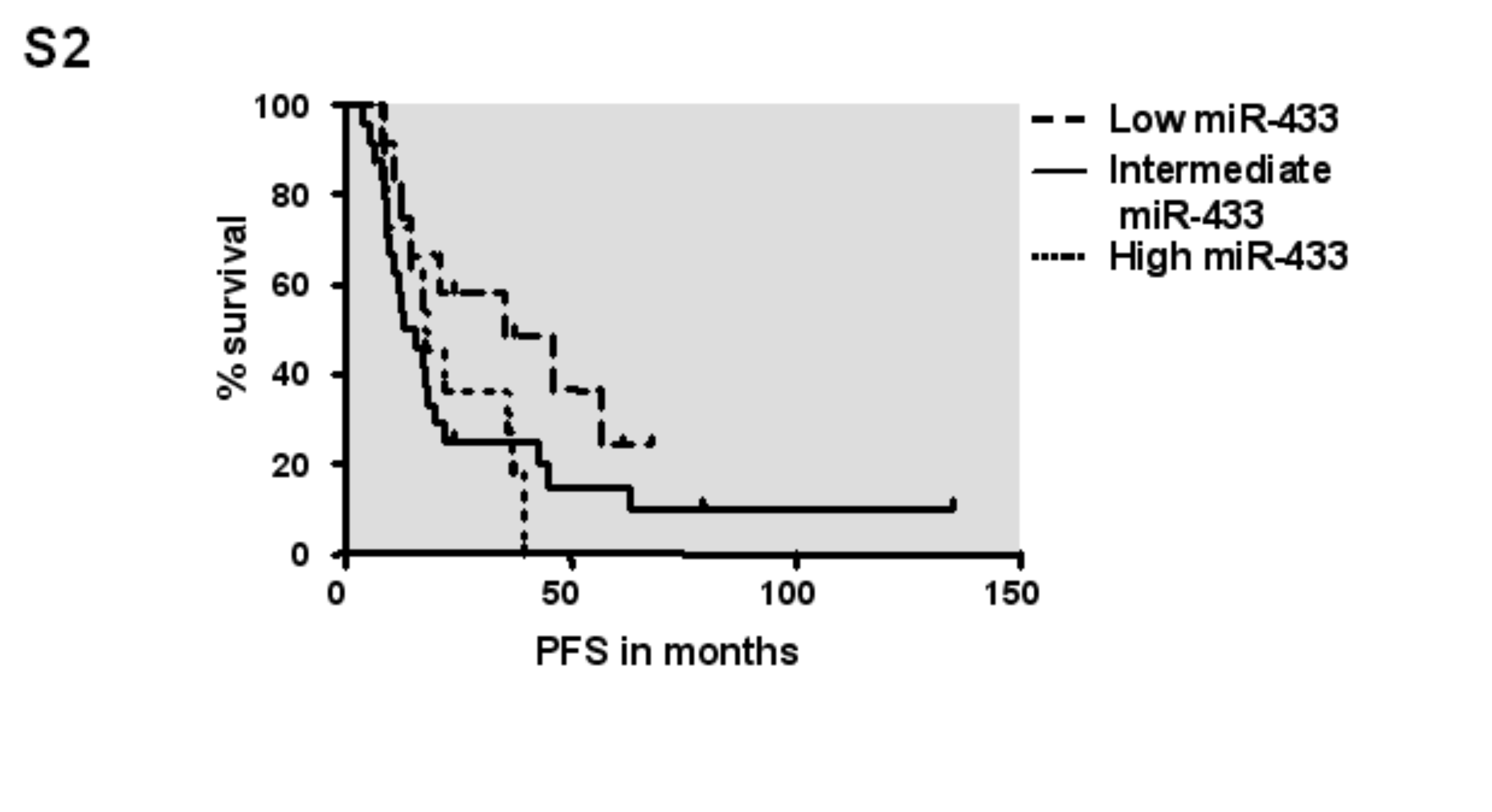

Supplement: Supplementary file 2 [file path0226-0746-SD2.tif]
