# Supplementary material for: Low MAD2 expression levels associate with reduced progression-free survival in patients with high-grade serous epithelial ovarian cancer
Source: J Pathol. 2012 Jan 17;226(5):746–55. doi: 10.1002/path.3035 (PMC3593171; doi:10.1002/path.3035)
Supplement: Supplementary file 3 [file path0226-0746-SD3.doc]

**Table S1. Clinicopathological details of ovarian specimens**

| **Full-face sections (patient cohort 1)** | | | |  | **Tissue microarray sections (patient cohort 2)** | | | |
| --- | --- | --- | --- | --- | --- | --- | --- | --- |
| **Tumour type** | **Stage** | **Grade** | **No.** |  | **Tumour type** | **Stage** | **Grade** | **No.** |
| Serous adenocarcinoma | I | 3 | 3 |  | Serous adenocarcinoma | I | 2 | 1 |
| Serous adenocarcinoma | II | 3 | 3 |  | Serous adenocarcinoma | I | 3 | 3 |
| Serous adenocarcinoma | III | 2 | 2 |  | Serous adenocarcinoma | I | 4 | 1 |
| Serous adenocarcinoma | IIIA | 3 | 1 |  | Serous adenocarcinoma | II | 2 | 2 |
| Serous adenocarcinoma | IIIB | 2 | 1 |  | Serous adenocarcinoma | II | 3 | 11 |
| Serous adenocarcinoma | IIIB | 3 | 1 |  | Serous adenocarcinoma | III | 2 | 1 |
| Serous adenocarcinoma | IIIC | 2 | 10 |  | Serous adenocarcinoma | III | 3 | 18 |
| Serous adenocarcinoma | IIIC | 3 | 16 |  |  |  |  |  |
| Serous adenocarcinoma | IIIC | ND | 3 |  |  |  |  |  |
| Serous adenocarcinoma | IV | 3 | 4 |  |  |  |  |  |
| Serous adenocarcinoma | IV | 2 | 1 |  |  |  |  |  |
|  |  |  |  |  |  |  |  |  |
| Total |  |  | 45 |  | Total |  |  | 37 |

ND, not documented.
