# Supplementary material for: Low MAD2 expression levels associate with reduced progression-free survival in patients with high-grade serous epithelial ovarian cancer
Source: J Pathol. 2012 Jan 17;226(5):746–55. doi: 10.1002/path.3035 (PMC3593171; doi:10.1002/path.3035)
Supplement: Supplementary file 4 [file path0226-0746-SD4.doc]

**Table S2.** Sequence of primers for cloning the MAD2 3′ UTR

| **Gene name** | **Primer sequence** | **Accession No.** | **Location on transcript**  **(bp)** | **Size** |
| --- | --- | --- | --- | --- |
| MAD2 | F: 5′-GGCCACTAGTCTACTACAATCCACAAAGTA-3′  R: 5′-GGCCAAGCTTAACAAACTTAACTTTATTTC-3′ | NM_002358 | 661–1381 | ~700 |
